# Spotlight On Early-career Researchers: an interview with Verena Ruprecht

**DOI:** 10.1038/s42003-019-0597-x

**Published:** 2019-10-08

## Abstract

Verena Ruprecht is a group Leader in the Cell & Developmental Biology program at the Centre for Genomic Regulation (CRG) in Barcelona. She studies organismal development using zebrafish as a system, taking an interdisciplinary approach involving cell and molecular biology, imaging and mathematical tools to obtain information on the scale of molecules, cells, and tissues. We have asked Dr. Ruprecht about her research and thoughts about the future of her field as part our series on early-career researchers.

**Figure 1:**
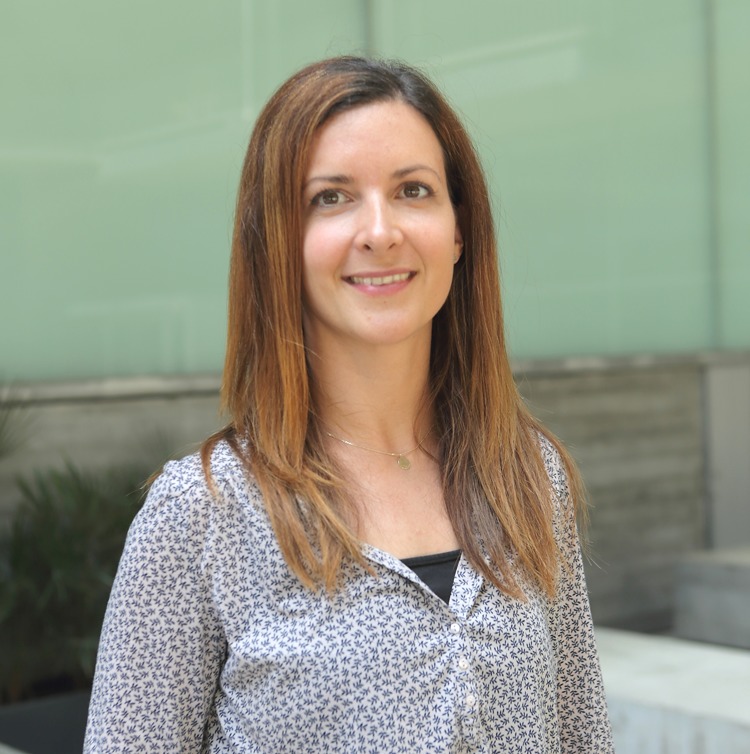
Verena Ruprecht

Please tell us about your research interests

Our lab is interested in understanding how an embryo builds its shape and functional tissue organization. We approach this fundamental question of how a single fertilized cell develops into a full organism from a multidisciplinary angle combining concepts and methods from physics and biology. We are specifically studying how single cell dynamics, such as cell shape changes, polarization, and motility are regulated and coordinated with cell fate specification programs to drive large-scale tissue reorganization. A key interest of our lab is to understand how mechanical forces and the physical tissue environment influence single cell dynamics and collective organization principles. This requires an understanding of how individual cells can sense and transmit mechanical forces and collectively interact to build functional tissue modules. Furthermore, we are interested how robustness of developmental processes is guaranteed in a noisy and error-prone tissue environment. As these questions are often difficult to exclusively assess in the in vivo context, we also make use of engineering approaches that allow us to dissect the in vivo system and reconstitute cellular dynamics and multi-cellular self-organization in simplified biomimetic ex vivo environments. These minimalistic assays further offer the opportunity to capture dynamic molecular processes in cells via cutting-edge imaging methods such as live cell superresolution imaging. This, for example, gives us the opportunity to visualize cytoskeletal motor protein activity at the single molecule level in live cells and to establish models on how mechanosensitive signal transduction pathways shape cytoskeletal architecture and cellular morphodynamics. The exciting and challenging part of our research is to integrate quantitative information from multiple scales and to build mechanistic models of single and multi-cellular organization principles. This is often done as a collaborative effort with theoretical physicists and team members coming from different scientific backgrounds, an enriching experience that allows us to approach scientific problems from multiple perspectives.

Why did you choose to be a scientist?

I was an incredibly curious child with broad interests. I simply enjoyed learning and exploring and somehow it naturally led me to pursue these interests further. In school, in my hometown of Klagenfurt, a quaint city in the very south of Austria, I became interested in mathematics and later decided to study physics. However, I already knew by then that I was more interested in understanding living systems rather than dead matter. This led me to choose the branch of biophysics during my studies and I became very fascinated by the emerging field of single molecule fluorescence microscopy, a technique that allowed for directly imaging the dynamics of individual proteins and lipids in live cells. I decided to pursue a Ph.D. in this research area, and it strongly shaped my passion for visualizing cellular dynamics, quantitative image processing, and modeling. From there, I became very interested in bridging the molecular stochastic view of the cell with physiological cellular processes. I was fortunate to get the opportunity to work jointly between the cell biology lab of Michael Sixt and the developmental biology lab run by Carl-Philipp Heisenberg during my postdoctoral studies. This opened the possibility to approach biological questions from a broad methodological and cross-disciplinary perspective. In our lab, we are now combining methods from physics and biology in combination with advanced fluorescence imaging that remains an exciting tool for visualization and discovery in biology.

What are your predictions for your field in the near future?

Methodological advances strongly shape the progress of the research field. Evolving techniques such as single cell sequencing and multi-scale imaging have opened an enormous amount of previously unavailable datasets to interrogate biological mechanisms and function. Novel molecular biology tools as Crispr/Cas9 or optogenetic tools offer new ways for precise and spatio-temporally controlled manipulation of biological systems. Also, new bottom-up approaches based on in vitro reconstitution and bioengineering methods will reveal the minimal components and requirements for single and multi-cellular self-organization and biological function. The increasing complexity of these emerging technologies along with the handling of large datasets and their interpretation will likely promote more interdisciplinary efforts and connect biological sciences with other previously more distant fields as physics, mathematics, and engineering.

Can you speak of any challenges that you have overcome?

Being a scientist can be a wonderful and fulfilling profession but is also associated to many challenges. Disappointments lurk on many corners, may it be slow progress or failures in experiments, rejections of articles, and funding proposals, or highly competitive career perspectives. In many cases, results and success are almost never immediate and it requires patience and perseverance to follow a scientific path. Also, international mobility is nowadays highly demanded and can lead to complications when settling in a foreign country and moving with a partner or family. In combination with a time-demanding job this often poses challenges to time-management and work–life balance. It helped me to embrace this journey as an opportunity to constantly learn both on the scientific and personal level and to reflect on core values, priorities, and important pillars of your personal life. More than ever before I now appreciate simple things that allow me to recharge my batteries, I enjoy going for a bike tour and hiking in the mountains and spending some precious time with my family and friends.

What advice would you give to your younger self?

Many mistakes that I have made were essential to learn and develop further as a person and scientist. In that aspect I would like to remind myself that it is most important not to be afraid of mistakes and failures as they naturally make you grow. I try to balance criticism and being kind with myself, and seeing positive and motivating aspects in difficult situations makes it easier for me to celebrate the good times and take along the best from challenging times.

What is the funniest or most memorable thing that has happened to you in the lab?

I am glad to remember many exciting and joyful events in the lab, many of them being connected to simple things that just revealed their relevance much later—from sharing the success of an experiment with an emotional hug, spontaneous lab parties to night-long discussions in the microscopy lab. I have met many great colleagues with whom I had both intense scientific interactions and maintain long-time friendships. In that sense, the lab environment for me is a special place, where both the rational minds and passionate souls of scientists meet and new exciting things are created.

*This interview was conducted by Senior Editor Christina Karlsson Rosenthal*

